# A nomogram based on LASSO-logistic regression for predicting tocilizumab-associated hypofibrinogenemia in patients with rheumatic diseases

**DOI:** 10.3389/fphar.2026.1861187

**Published:** 2026-07-31

**Authors:** Chunmei Dai, Yiting Wang, Yilei Chen, Jingwen Xie, Zhuoling Zheng, Jianlin Huang, Xiaoyan Li

**Affiliations:** 1 Department of Pharmacy, The Sixth Affiliated Hospital, Sun Yat-Sen University, Guangzhou, Guangdong, China; 2 Biomedical Innovation Center, The Sixth Affiliated Hospital, Sun Yat-sen University, Guangzhou, Guangdong, China; 3 School of Pharmacy and Clinical Pharmacy (School of Integrated Pharmacy), Guangdong Pharmaceutical University, Guangzhou, Guangdong, China; 4 Department of Rheumatology, The Sixth Affiliated Hospital, Sun Yat-Sen University, Guangzhou, Guangdong, China

**Keywords:** clinical prediction model, hypofibrinogenemia, nomogram, rheumatic diseases, tocilizumab

## Abstract

**Introduction:**

Tocilizumab (TCZ), an IL-6 receptor inhibitor used in rheumatic diseases, can suppress fibrinogen synthesis and cause hypofibrinogenemia, yet its incidence, risk factors and predictive tools remain incompletely characterized. This study determined the incidence of TCZ-associated hypofibrinogenemia and developed a nomogram-based prediction model for individualized risk stratification.

**Methods:**

This retrospective study was conducted at the Sixth Affiliated Hospital of Sun Yat-sen University, enrolling 121 patients with rheumatic diseases who received TCZ between 1 January 2019 and 31 December 2025 and had at least one fibrinogen measurement. Hypofibrinogenemia was defined as a plasma fibrinogen concentration below 2.0 g/L at any point during treatment. Hypofibrinogenemia was defined as a plasma fibrinogen concentration below 2.0 g/L at any point during treatment. All candidate predictors were entered into Least Absolute Shrinkage and Selection Operator (LASSO) logistic regression with 10-fold cross-validation under the λ.1se criterion, and the retained candidates were examined in multivariable logistic regression. The resulting coefficients were formulated into a nomogram, whose performance was assessed in terms of discrimination by the area under the receiver operating characteristic curve (AUC), calibration by the Hosmer-Lemeshow test and bias-corrected calibration curves, internal validity through 1000-replicate bootstrap resampling, clinical utility via decision curve analysis and clinical impact curves, and predictor-level interpretability through SHapley Additive exPlanations (SHAP) decomposition.

**Results:**

Hypofibrinogenemia occurred in 39 of 121 patients (32.23%). Independent predictors were higher BMI, lower IgA, lower platelet count, and treatment with Actemra® rather than Tofidence®. The nomogram showed acceptable discrimination (AUC = 0.795, 95% CI: 0.716–0.873; bootstrap-corrected AUC = 0.782, 95% CI: 0.751–0.795), good calibration (Hosmer-Lemeshow *P* = 0.835; Brier score = 0.170), and positive net benefit on decision curve analysis. SHAP analysis identified platelet count as the dominant contributor, followed by TCZ brand, IgA and BMI.

**Conclusion:**

Hypofibrinogenemia occurred in one-third of TCZ-treated rheumatic patients. Higher BMI, lower IgA, lower platelet count and use of the Actemra® were independently associated with increased risk. The nomogram offers a preliminary tool for risk stratification, but requires external validation before clinical use.

## 1 Introduction

Tocilizumab (TCZ), a recombinant humanized monoclonal antibody that inhibits both soluble and membrane-bound interleukin-6 (IL-6) receptors, has been widely used in the treatment of various inflammatory rheumatic diseases. TCZ has demonstrated well-established efficacy across several immune-mediated diseases, including rheumatoid arthritis (RA), giant cell arteritis (GCA), systemic juvenile idiopathic arthritis (sJIA) and adult-onset Still’s disease (AOSD) (Sheppard et al., 2017; Tanaka et al., 2018; Sota et al., 2022; Brunner et al., 2024). Beyond rheumatology, recent studies have also explored its role in acute ST-segment elevation myocardial infarction (STEMI), where IL-6 receptor inhibition improved myocardial salvage index (Huse et al., 2025), and in acute ischaemic stroke undergoing endovascular treatment, where TCZ reduced infarct volume growth (Chu et al., 2026). Furthermore, case reports have documented the successful use of TCZ in managing severe hypersensitivity reactions associated with cytokine release following oxaliplatin infusion (Liao and Jiang, 2024). By blocking the IL-6 signaling pathway, TCZ effectively mitigates the systemic inflammatory response, leading to significant improvements in clinical symptoms and laboratory markers of inflammation. Beyond its impact on classic inflammatory markers like C-reactive protein (CRP), TCZ exerts a profound effect on the coagulation cascade, particularly on fibrinogen (FIB) levels. As an acute-phase reactant predominantly synthesized by hepatocytes under the transcriptional control of IL-6, fibrinogen levels typically decrease during TCZ treatment (Woods et al., 2000; Mokuda et al., 2013). While this reduction is often expected and remains within the normal range, a growing body of evidence indicates that some patients may develop hypofibrinogenemia, defined as plasma fibrinogen levels dropping below 2.0 g/L (Levy and Goodnough, 2015). Notably, TCZ-associated hypofibrinogenemia has been observed across diverse clinical settings, including COVID-19 treatment (Gao et al., 2025) and CAR-T cell therapy (Perl et al., 2024), suggesting that this adverse event is not limited to rheumatic diseases but represents a class effect of IL-6 blockade. TCZ-associated hypofibrinogenemia has raised clinical concerns due to the potential risk of bleeding complications, ranging from mild ecchymosis and gingival bleeding to more severe hemorrhagic events (Matsuoka et al., 2012; Martis et al., 2017; Imamura et al., 2018; Üsküdar Cansu et al., 2019).

The reported incidence of TCZ-associated hypofibrinogenemia varies considerably across studies. In adult patients with rheumatic diseases, the frequency has been observed to range from 29% to approximately 46.6% (Okano et al., 2016; An et al., 2024; Yıldırım et al., 2024). Notably, a significantly higher incidence of 76.47% was reported in a cohort of patients with sJIA, suggesting that the underlying disease and patient age may influence susceptibility (He et al., 2023). Pharmacovigilance data from the FDA Adverse Event Reporting System (FAERS) have also identified potential safety signals related to TCZ use during pregnancy, including neonatal death, though these associations require confirmation in future clinical studies (Romao et al., 2024). Clinically, TCZ-associated hypofibrinogenemia often occurs within the first 3 months of treatment, frequently after the initial 1 to 4 doses (Souri et al., 2016; An et al., 2024). Despite increasing recognition of this adverse event, there is no consensus on the necessity of routine fibrinogen monitoring or standardized management strategies for patients who develop this condition (Souri et al., 2016; He et al., 2023).

However, most previous studies have focused on acute inflammatory conditions (e.g., COVID-19, CAR-T therapy) or a single rheumatic subtype (e.g., RA), leaving the risk profile in mixed chronic rheumatic diseases largely unexplored. Furthermore, existing prediction models have not incorporated easily accessible laboratory parameters such as IgA and PLT, nor have they compared the safety profiles of different TCZ products (originator vs. biosimilar). Therefore, this study aims to investigate the incidence and clinical characteristics of TCZ-associated hypofibrinogenemia in a cohort of patients with rheumatic diseases and to identify independent risk factors for its development, thereby providing evidence-based guidance for clinical monitoring and management.

## 2 Materials and methods

### 2.1 Study design

This was a real-world, retrospective, observational study conducted at the Sixth Affiliated Hospital of Sun Yat-sen University. A total of 121 patients who received TCZ between 1 January 2019 and 31 December 2025 were enrolled.

The diagnosis of each disease was established according to the following classification criteria: rheumatoid arthritis (RA) met the 2010 ACR/EULAR criteria (Aletaha et al., 2010); Takayasu arteritis (TA) met the 1990 ACR criteria (Arend et al., 1990); dermatomyositis (DM) met the 1975 Bohan-Peter criteria (Bohan and Peter, 1975); adult-onset Still’s disease (AOSD) met the 1992 Yamaguchi criteria (Yamaguchi et al., 1992).

Patients were eligible if they (1) were aged ≥18 years with a rheumatic disease diagnosed according to current internationally accepted classification criteria; (2) received TCZ in accordance with the prescribing information (intravenous 8 mg/kg every 4 weeks) and had completed at least two consecutive doses, or reached a study endpoint before the second dose, whichever came first; (3) had a baseline FIB measurement within 7 days before the first TCZ administration and at least two further FIB measurements during the study period; and (4) had at least 6 months of follow-up, or follow-up until the occurrence of a study endpoint, whichever came first.

Patients were excluded for any of the following: (1) absence of a baseline FIB value, or fewer than two on-treatment FIB measurements; (2) baseline FIB <2.0 g/L prior to TCZ treatment; (3) pregnancy or lactation; (4) pre-existing hematological disorders (e.g., leukemia, lymphoma, myelodysplastic syndrome, hemophilia, or other congenital or acquired coagulation disorders); (5) clinically significant hepatic disease, defined as cirrhosis (Child–Pugh class B or C), chronic active hepatitis, ALT or AST > 3 × the upper limit of normal (ULN), or total bilirubin > 2 × ULN; (6) major surgery (a procedure under general anesthesia with estimated blood loss >500 mL) within 6 weeks before, or at any time during, the study period; (7) active severe infection, sepsis, or laboratory-confirmed disseminated intravascular coagulation at baseline or during follow-up; (8) active malignancy; (9) prior TCZ exposure, or exposure to any other IL-6 pathway inhibitor (e.g., siltuximab, sarilumab) within 3 months before TCZ initiation; (10) concomitant use, at baseline or at any time during the study period, of agents known to lower fibrinogen or materially alter coagulation, including anticoagulants (heparin, warfarin, direct oral anticoagulants), therapeutic-dose antiplatelet agents, asparaginase, bevacizumab, thrombolytic agents, and antibiotics with an established association with hypofibrinogenemia (e.g., tigecycline, cefoperazone, linezolid, daptomycin); (11) incomplete key clinical records, including baseline demographics, comorbidities, or concomitant medications.

Each suspected adverse drug reaction was independently reviewed by two senior clinical pharmacists, with discrepancies resolved by consensus or third-party adjudication. Causality was graded using the Naranjo Adverse Drug Reaction Probability Scale, and reactions with a score ≥5 were classified as probable (Agbabiaka et al., 2008).

All procedures involving human participants in this study were conducted in accordance with the ethical standards of the institutional and/or national research committee and with the 1964 Helsinki Declaration, including its later amendments or comparable ethical standards. The study protocol was approved by the Ethics Committee of the Sixth Affiliated Hospital, Sun Yat-sen University (No. 2026ZSLYEC-124). As this was a retrospective observational study, informed consent was waived.

### 2.2 Data collection

Data were retrospectively extracted from the hospital information system. The collected covariates were grouped into the following categories: (1) Demographic and baseline characteristics: sex, age (years), body mass index (BMI, kg/m^2^), disease duration (years), smoking history, drinking history and comorbidities including diabetes, hypertension, coronary heart disease and hyperlipidemia. (2) Disease-specific characteristics: rheumatic disease diagnosis, tender joint count, swollen joint count, C-reactive protein (CRP, mg/L), erythrocyte sedimentation rate (ESR, mm/h), rheumatoid factor (RF, IU/mL) and anti-cyclic citrullinated peptide (anti-CCP, U/mL). (3) Treatment-related variables: TCZ brand name (Tofidence® or Actemra®). (4) Laboratory parameter-including the following subcategories: Coagulation profile including fibrinogen (FIB, g/L), activated partial thromboplastin time (APTT, s), thrombin time (TT, s) and international normalized ratio (INR). Liver and kidney function tests: alanine aminotransferase (ALT, U/L), aspartate aminotransferase (AST, U/L), gamma-glutamyl transferase (GGT, U/L), total bilirubin (TBIL, μmol/L), albumin (ALB, g/L) and creatinine (Cr, μmol/L). Lipid profile: triglycerides (TG, mmol/L) and total cholesterol (TC, mmol/L). Immunoglobulins and complements: immunoglobulin G (IgG, g/L), immunoglobulin A (IgA, g/L), immunoglobulin M (IgM, g/L), complement C3 (g/L) and complement C4 (g/L). Complete blood count: white blood cell count (WBC, ×10^9^/L), red blood cell count (RBC, ×10^12^/L), hemoglobin (HGB, g/L) and platelet count (PLT, ×10^9^/L). Other laboratory tests: glucose (GLU, mmol/L).

Patients were subsequently stratified into two groups based on the occurrence of hypofibrinogenemia during treatment: the hypofibrinogenemia group (fibrinogen level <2.0 g/L) and the normal fibrinogen group (fibrinogen level ≥2.0 g/L).

### 2.3 Statistical analysis

SPSS (version 27.0) and R (version 4.5.0) served as the analytical platform throughout this study. Normality of continuous variables was assessed using the Kolmogorov-Smirnov test. Continuous variables conforming to a normal distribution are reported as mean ± standard deviation (SD) and contrasted between groups via the independent-samples t-test; those deviating from normality are expressed as median (interquartile range, IQR) and evaluated using the Mann–Whitney U test. Categorical data are presented as counts with proportions, and between-group differences were examined by Pearson’s chi-square test, with Fisher’s exact test substituted whenever an expected cell frequency fell below 5.

Variables with a missingness rate greater than 30% were excluded prior to imputation, and missing values in the remaining continuous variables (Supplementary Figure S1; Supplementary Table S1) were subsequently imputed using the missRanger package in R based on a random forest algorithm (Stekhoven and Bühlmann, 2012; Hou et al., 2025). All predictors were carried forward into least absolute shrinkage and selection operator (LASSO) logistic regression with 10-fold cross-validation, and the optimal regularisation parameter was defined at λ.1se to favour a parsimonious predictor set. The selected variables were then incorporated into a multivariable logistic regression model, and the estimated coefficients were reformulated as a nomogram to support patient-level probability estimation.

Bootstrap resampling with 1000 repeats was used for internal validation. Model discrimination was evaluated by area under the receiver operating characteristic curve (AUC). The DeLong test was applied to compare the apparent AUC with the bootstrap validated AUC, and *P* ≥ 0.05 was considered to indicate the absence of substantial overfitting. Calibration was evaluated by plotting calibration curves and performing the Hosmer-Lemeshow test to examine the predictive accuracy and fit of the nomogram. A Hosmer-Lemeshow *P* value >0.05 was considered to indicate acceptable agreement between predicted and observed outcomes. Clinical usefulness was assessed by decision curve analysis (DCA) through comparison of the net benefit of the model with the treat all and treat none strategies. Clinical impact curve (CIC) was used to illustrate the expected number of true positive cases among 100 patients across different threshold probabilities. The contribution of every predictor to the model’s output was measured using SHAP values, calculated at both the population and individual levels, in order to enhance model interpretability. All hypothesis tests were two-sided, with statistical significance defined as *P* < 0.05.

## 3 Results

### 3.1 Patient characteristics

**FIGURE 1:**
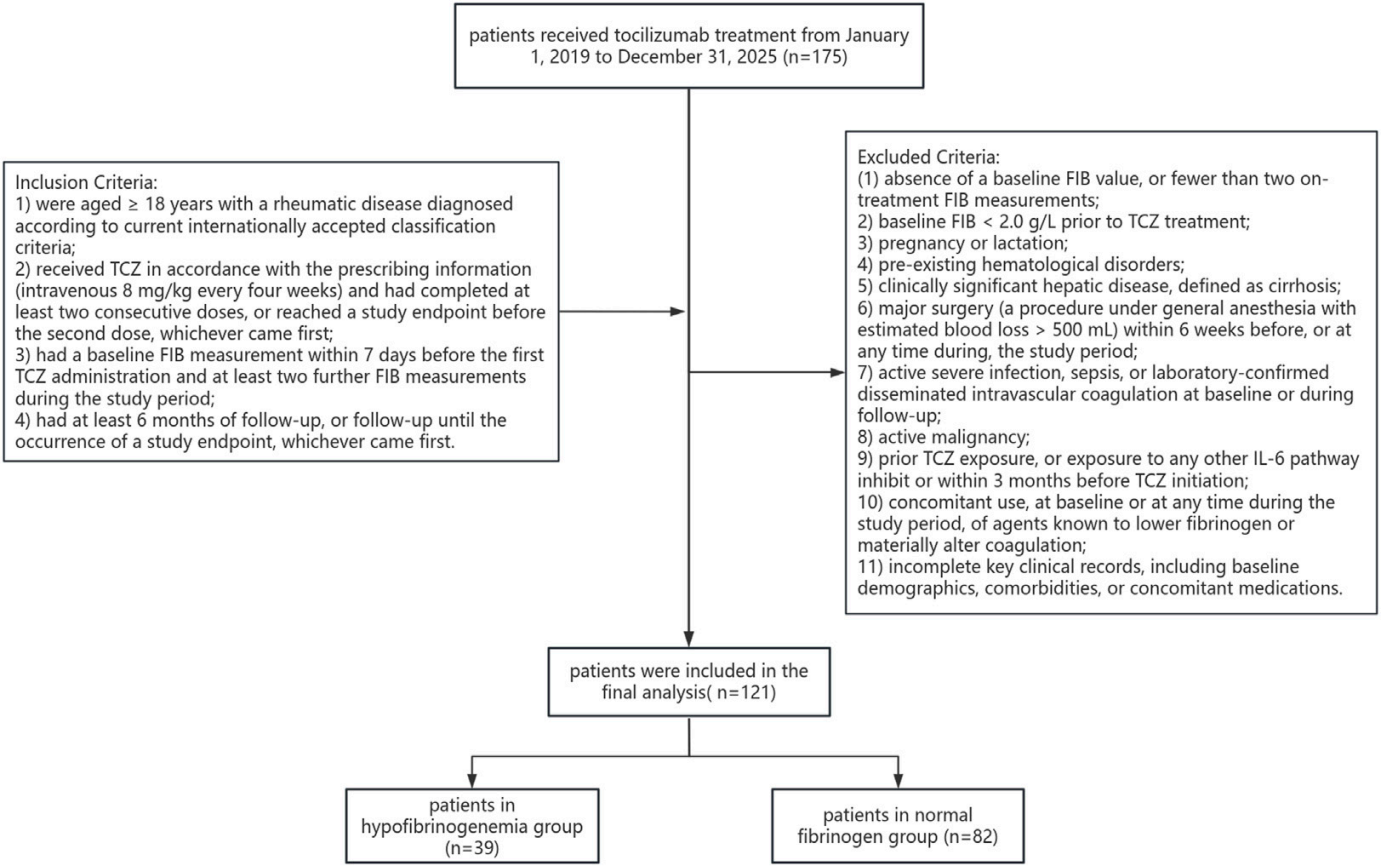
Flow chart of the study population.

A total of 175 patients treated with TCZ between 1 January 2019 and 31 December 2025 were initially screened, of whom 121 patients met the eligibility criteria and were included in the final analysis (Figure 1). During a follow-up period of at least 6 months, 39 patients developed TCZ-associated hypofibrinogenemia, whereas 82 patients did not. Baseline comparisons demonstrated that patients who developed hypofibrinogenemia had a significantly higher BMI than those without this complication (*P* < 0.05). Significant differences were also observed in TCZ drug dosage and drug brand distribution (all *P* < 0.05). In laboratory parameters, FIB, PLT, IgA and ESR all differed significantly between the two groups (all *P* < 0.05). In contrast, a broad range of clinical and biochemical variables showed no appreciable between-group differences, including tender and swollen joint counts, disease duration, CRP, RF, anti-CCP, APTT, TT, INR, TBIL, ALB, ALT, AST, Cr, IgG, IgM, sex, disease subtype, comorbid conditions, smoking history, alcohol history and concomitant medication use (all *P* > 0.05). The demographics and baseline characteristics were shown in Table 1.

**TABLE 1:** Baseline characteristics of patients with and without TCZ-associated hypofibrinogenemia.

Variables	Total (n = 121)	FIB-normal group (n = 82)	FIB-reduced group (n = 39)	*P* value
Sex, n (%)	​	​	​	0.114
Female	97 (80.17)	62 (75.61)	35 (89.74)	​
Male	24 (19.83)	20 (24.39)	4 (10.26)	​
Age, years, mean ± SD	59.40 ± 13.25	60.89 ± 13.18	56.28 ± 13.04	0.074
BMI, kg/m^2^, mean ± SD	22.04 ± 3.65	21.46 ± 3.74	23.24 ± 3.17	0.008
Disease duration, years, median (IQR)	9.00 (4.67, 12.08)	9.12 (4.35, 10.88)	9.00 (5.46, 13.04)	0.538
Rheumatic diseases, n (%)	​	​	​	0.593
RA, n (%)	117 (96.69)	80 (97.56)	37 (94.87)	​
AOSD, n (%)	2 (1.65)	1 (1.22)	1 (2.56)	​
DM, n (%)	1 (0.83)	0 (0.00)	1 (2.56)	​
TA, n (%)	1 (0.83)	1 (1.22)	0 (0.00)	​
Diabetes, n (%)	14 (11.57)	8 (9.76)	6 (15.38)	0.376
Hypertension, n (%)	35 (28.93)	24 (29.27)	11 (28.21)	1.000
Coronary heart disease, n (%)	12 (9.92)	9 (10.98)	3 (7.69)	0.750
Hyperlipidemia, n (%)	17 (14.05)	9 (10.98)	8 (20.51)	0.258
Smoking history, n (%)	10 (8.26)	9 (10.98)	1 (2.56)	0.165
Drinking history, n (%)	4 (3.31)	3 (3.66)	1 (2.56)	1.000
Drug dosage	400.00 (400.00, 480.00)	400.00 (400.00, 480.00)	480.00 (400.00, 480.00)	0.031
Drug brand name, n (%)				
Tofidence®	85 (70.25)	64 (78.05)	21 (53.85)	0.012
Actemra®	36 (29.75)	18 (21.95)	18 (46.15)	​
Tender joint count, median (IQR)	5.00 (2.00, 12.00)	4.00 (2.00, 11.00)	6.00 (1.50, 17.50)	0.252
Swollen joint count, median (IQR)	3.00 (1.00, 10.00)	4.50 (2.00, 10.75)	2.00 (0.50, 9.00)	0.357
WBC, ×10^9^/L, mean ± SD	6.48 ± 2.41	6.46 ± 2.31	6.53 ± 2.63	0.880
RBC, ×10^12^/L, mean ± SD	3.81 ± 0.62	3.84 ± 0.62	3.76 ± 0.64	0.540
HGB, g/L, mean ± SD	104.43 ± 18.44	104.33 ± 17.45	104.64 ± 20.61	0.935
PLT, ×10^9^/L, mean ± SD	287.12 ± 110.88	308.26 ± 115.45	242.69 ± 86.18	0.001
CRP, mg/L, median (IQR)	15.54 (5.53, 49.17)	15.69 (6.04, 53.40)	15.54 (4.15, 38.79)	0.585
ESR, mm/h, mean ± SD	48.58 ± 36.35	54.11 ± 38.39	40.05 ± 29.92	0.030
RF, IU/mL, median (IQR)	106.13 (26.04, 292.43)	140.73 (37.98, 304.38)	57.43 (20.71, 242.00)	0.110
Anti-CCP, U/mL, median (IQR)	309.20 (45.10, 1182.86)	265.27 (40.62, 1040.63)	557.12 (119.50, 2000.00)	0.093
FIB, g/L, mean ± SD	3.96 ± 1.12	4.11 ± 1.24	3.61 ± 0.72	0.005
APTT, s, mean ± SD	30.49 ± 3.95	30.58 ± 4.09	30.31 ± 3.69	0.721
TT, s, median (IQR)	14.70 (13.90, 15.50)	14.75 (13.93, 15.60)	14.60 (13.90, 15.40)	0.538
INR, median (IQR)	0.99 (0.95, 1.06)	0.99 (0.95, 1.06)	1.01 (0.96, 1.06)	0.368
TBIL, μmol/L, mean ± SD	8.93 ± 3.36	9.11 ± 3.02	8.54 ± 3.99	0.433
TG, mmol/L, median (IQR)	0.90 (0.69, 1.20)	0.86 (0.67, 1.14)	1.02 (0.78, 1.29)	0.068
TC, mmol/L, mean ± SD	4.25 ± 0.96	4.24 ± 0.95	4.27 ± 1.00	0.871
GGT, U/L, median (IQR)	18.11 (13.15, 27.14)	19.36 (13.20, 26.66)	16.62 (12.23, 29.66)	0.850
ALB, g/L, mean ± SD	34.92 ± 4.85	34.61 ± 4.99	35.59 ± 4.52	0.282
ALT, U/L, median (IQR)	15.00 (11.06, 20.62)	15.44 (11.56, 19.96)	13.59 (10.54, 22.80)	0.694
AST, U/L, median (IQR)	18.10 (15.08, 22.96)	18.07 (15.21, 23.29)	18.41 (14.91, 22.03)	0.765
Cr, μmol/L, median (IQR)	64.37 (54.52, 80.44)	63.89 (53.17, 82.04)	64.40 (56.59, 73.84)	0.967
IgG, g/L, mean ± SD	13.38 ± 4.63	13.75 ± 5.03	12.60 ± 3.59	0.154
IgA, g/L, mean ± SD	2.82 ± 1.17	3.07 ± 1.19	2.30 ± 0.93	<0.001
IgM, g/L, median (IQR)	1.20 (0.88, 1.52)	1.20 (0.95, 1.48)	1.12 (0.81, 1.60)	0.635
C3, g/L, mean ± SD	1.18 ± 0.25	1.20 ± 0.24	1.14 ± 0.27	0.213
C4, g/L, mean ± SD	0.28 ± 0.13	0.28 ± 0.10	0.29 ± 0.17	0.931
GLU, mmol/L, median (IQR)	4.82 (4.33, 5.50)	4.78 (4.29, 5.48)	4.93 (4.41, 5.57)	0.633
Concomitant medication, n (%)				
Methotrexate	58 (47.93)	35 (42.68)	23 (58.97)	0.138
Hydroxychloroquine	90 (74.38)	64 (78.05)	26 (66.67)	0.264
Low dose glucocorticoids	51 (42.15)	34 (41.46)	17 (43.59)	0.981

TCZ, tocilizumab; FIB, fibrinogen; BMI, body mass index; RA, rheumatoid arthritis; AOSD, adult-onset Still’s disease; DM, dermatomyositis; TA, takayasu arteritis; WBC, white blood cell count; RBC, red blood cell count; HGB, hemoglobin; PLT, platelet count; CRP, C-reactive protein; ESR, erythrocyte sedimentation rate; RF, rheumatoid factor; Anti-CCP, anti-cyclic citrullinated peptide antibody; APTT, activated partial thromboplastin time; TT, thrombin time; INR, international normalized ratio; TBIL, total bilirubin; TG, triglycerides; TC, total cholesterol; GGT, gamma-glutamyl transferase; ALB, albumin; ALT, alanine aminotransferase; AST, aspartate aminotransferase; Cr, creatinine; IgG, immunoglobulin G; IgA, immunoglobulin A; IgM, immunoglobulin M; C3, complement C3; C4, complement C4; GLU, glucose.

In the overall population, the median time from TCZ initiation to onset of hypofibrinogenemia was 64 days (IQR 108), with a median of 2 infusions (IQR 3). The median fibrinogen nadir was 1.70 g/L (IQR 0.30), representing a mean reduction of 53.00% ± 11.28% from baseline. During recovery, fibrinogen rebounded above the normal range in 23 patients (58.97%), with a median time to rebound of 58 days (IQR 156.5). Fibrinogen supplementation was administered in 2 patients (5.13%) and no clinically significant bleeding events, including ecchymosis, gingival bleeding, petechiae, hematemesis or other overt hemorrhagic manifestations were observed. The FIB level and outcome measures in patients with TCZ-associated hypofibrinogenemia were shown in Table 2.

**TABLE 2:** FIB level and outcome measures in patients with TCZ-associated hypofibrinogenemia.

Variables	Total (n = 39)	RA (n = 37)	AOSD (n = 1)	DM (n = 1)
Time to onset, days, median (IQR)	64 (108)	65 (111)	3	1
Number of intravenous infusions before onset, times, median (IQR)	2 (3)	2 (4)	1	1
Fibrinogen nadir, g/L, median (IQR)	1.70 (0.30)	1.70 (0.26)	1.60	0.90
Fibrinogen reduction from baseline, %, mean ± SD	53.00 ± 11.28	52.58 ± 11.59	52.94	64.00
Fibrinogen rebound above normal, n (%)	23 (58.97)	22 (59.46)	1 (100.00)	0 (0.00)
Time to rebound to normal range, days, median (IQR)	58 (156.5)	68.5 (122)	9	0
Fibrinogen infusions, n (%)	2 (5.13)	1 (2.70)	0 (0.00)	1 (100.00)
TCZ discontinuation due to hypofibrinogenemia, n (%)	13 (33.33)	11 (29.73)	1 (100.00)	1 (100.00)

TCZ, tocilizumab; FIB, fibrinogen; RA, rheumatoid arthritis; AOSD, adult-onset Still’s disease; DM, dermatomyositis; Rebound is defined as the first return of fibrinogen level to above the lower limit of the normal range (≥2.0 g/L) after the occurrence of hypofibrinogenemia.

### 3.2 LASSO based variable selection and multivariable logistic regression analysis

**FIGURE 2:**
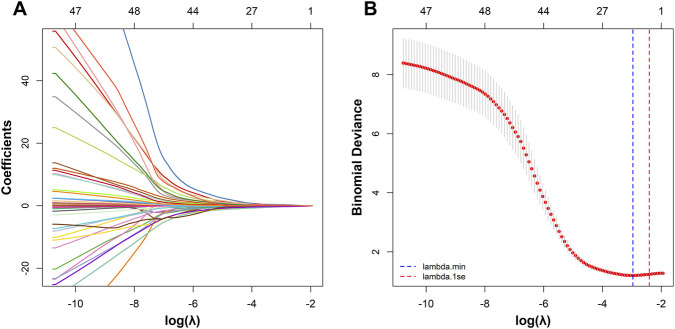
Least absolute shrinkage and selection operator (LASSO) logistic regression for predictor selection in the risk model of TCZ-associated hypofibrinogenemia: **(A)** coefficient trajectories of candidate variables across the log(λ) sequence; **(B)** cross-validation plot of binomial deviance for determination of the optimal penalty parameter (λ).

To account for potential multicollinearity among predictors and to avoid the selection bias introduced by univariate pre-screening, all candidate variables were entered into LASSO regression, which performs simultaneous coefficient shrinkage and variable selection. As shown in Figure 2A, with increasing penalization, the coefficients of candidate variables gradually shrank toward zero, indicating progressive exclusion of less informative predictors. The optimal penalty parameter was determined by cross validation (Figure 2B). Based on the lambda.1se criterion, the variables retained at this penalty level were selected for subsequent analysis and the corresponding coefficients are presented in Supplementary Table S2.

We incorporated the variables selected by LASSO into a multivariable logistic regression model. As presented in Table 3, BMI, PLT, IgA and TCZ brand remained independently associated with TCZ-associated hypofibrinogenemia after full adjustment.

**TABLE 3:** Multivariable logistic regression analysis for identifying independent predictors of TCZ-associated hypofibrinogenemia.

Variables	*β*	*SE*	*Wald χ* ^*2*^	OR	95% CI	*P*
BMI	0.158	0.067	5.632	1.172	1.172 (1.028, 1.335)	0.018
PLT	−0.007	0.003	7.100	0.993	0.993 (0.988, 0.998)	0.008
IgA	−0.522	0.224	5.430	0.593	0.593 (0.382, 0.920)	0.020
TCZ brand (Actemra®)	1.230	0.487	6.381	3.423	3.423 (1.318, 8.891)	0.012

IgA, immunoglobulin A; BMI, body mass index; PLT, platelet count; TCZ, tocilizumab.

Specifically, BMI was positively associated with the risk of hypofibrinogenemia, with each unit increase corresponding to greater odds of the outcome (OR = 1.172, 95% CI: 1.028–1.335, *P* = 0.018). Conversely, PLT exhibited a significant inverse relationship, such that higher platelet counts were associated with a reduced likelihood of developing hypofibrinogenemia (OR = 0.993, 95% CI: 0.988–0.998, *P* = 0.008). IgA similarly demonstrated an independent inverse association, with lower levels associated with increased odds of the event (OR = 0.593, 95% CI: 0.382–0.920, *P* = 0.020). Regarding TCZ formulation, use of Actemra® was identified as an independent predictor, conferring a markedly elevated risk relative to the comparator brand (OR = 3.423, 95% CI: 1.318–8.891, *P* = 0.012). Although TG was retained in the multivariable model following LASSO selection, neither reached statistical significance in the adjusted analysis (*P* > 0.05).

Collectively, these findings indicate that LASSO regression effectively narrowed the pool of candidate variables derived from univariable screening, preserving only those with the most meaningful predictive contribution. Subsequent multivariable logistic regression confirmed BMI, PLT, IgA and TCZ brand as independent predictors of TCZ-associated hypofibrinogenemia, providing a robust basis for their incorporation into the final risk prediction model.

### 3.3 Nomogram for individualized risk prediction

**FIGURE 3:**
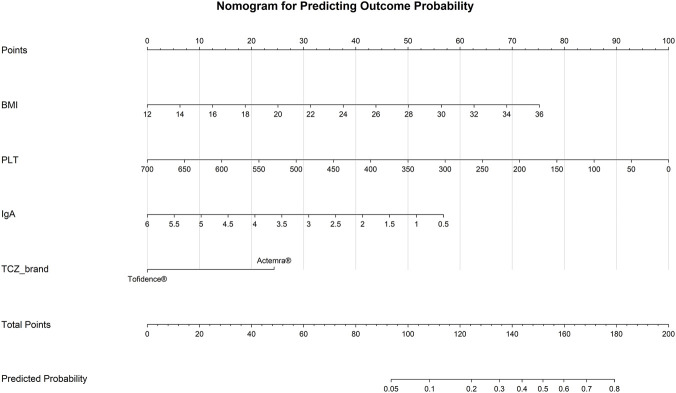
Nomogram predicting the risk of TCZ-associated hypofibrinogenemia.

A nomogram was constructed on the basis of the multivariable logistic regression model to estimate the individual risk of TCZ-associated hypofibrinogenemia (Figure 3). The model incorporated BMI, PLT, IgA and TCZ brand, all of which remained independent predictors in the multivariable analysis. Specifically, higher BMI was associated with an increased risk, whereas higher PLT and higher IgA were associated with a reduced risk. In addition, TCZ brand was significantly associated with the outcome, and the use of Actemra® corresponded to a higher predicted risk than Tofidence®. In the nomogram, each predictor was assigned a score according to its regression coefficient and the sum of these scores was translated into the estimated probability of hypofibrinogenemia. This nomogram provides an intuitive tool for individualized risk stratification and may assist in the early identification of patients at increased risk during TCZ treatment.

### 3.4 Distribution and flow of patients across key clinical risk strata in relation to TCZ-induced hypofibrinogenemia

**FIGURE 4:**
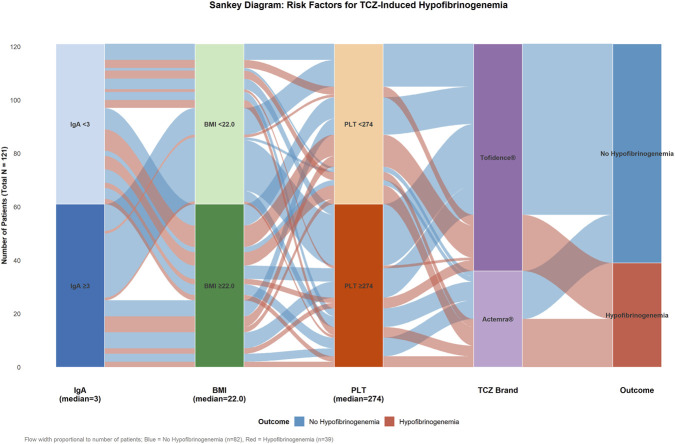
Sankey diagram illustrating risk stratification for TCZ-associated hypofibrinogenemia.

A Sankey diagram was constructed to depict the distribution of 121 patients across four stratified variables (IgA, BMI, PLT and TCZ brand) and their collective flow toward hypofibrinogenemia (Figure 4). Continuous variables were dichotomized at their respective median values: IgA (3.0 g/L), BMI (22.0 kg/m^2^) and PLT (274 × 10^9^/L). Among patients with lower IgA levels, a notably greater proportion of flow streams converged toward the hypofibrinogenemia node compared with those with higher IgA levels, suggesting that lower IgA were overrepresented among those who developed this complication. A comparable pattern emerged for BMI, with patients in the higher BMI stratum (≥22.0 kg/m^2^) showing wider flow bands directed toward the adverse outcome node relative to their lower-BMI counterparts. Conversely, patients with PLT below the median (<274 × 10^9^/L) displayed a disproportionate concentration of flow toward the hypofibrinogenemia endpoint, implying that lower baseline platelet counts conferred greater susceptibility to this complication. With respect to TCZ brand, patients receiving Actemra® exhibited broader flow bands directed toward the hypofibrinogenemia node than those receiving Tofidence®, reflecting a markedly higher proportion of affected individuals within the Actemra® subgroup. Taken together, the Sankey diagram provides an integrated visual summary of how these four clinical variables, both individually and in combination, channeled patients toward or away from TCZ-associated hypofibrinogenemia, reinforcing the multifactorial nature of this adverse drug reaction.

### 3.5 Model performance and clinical applicability

**FIGURE 5:**
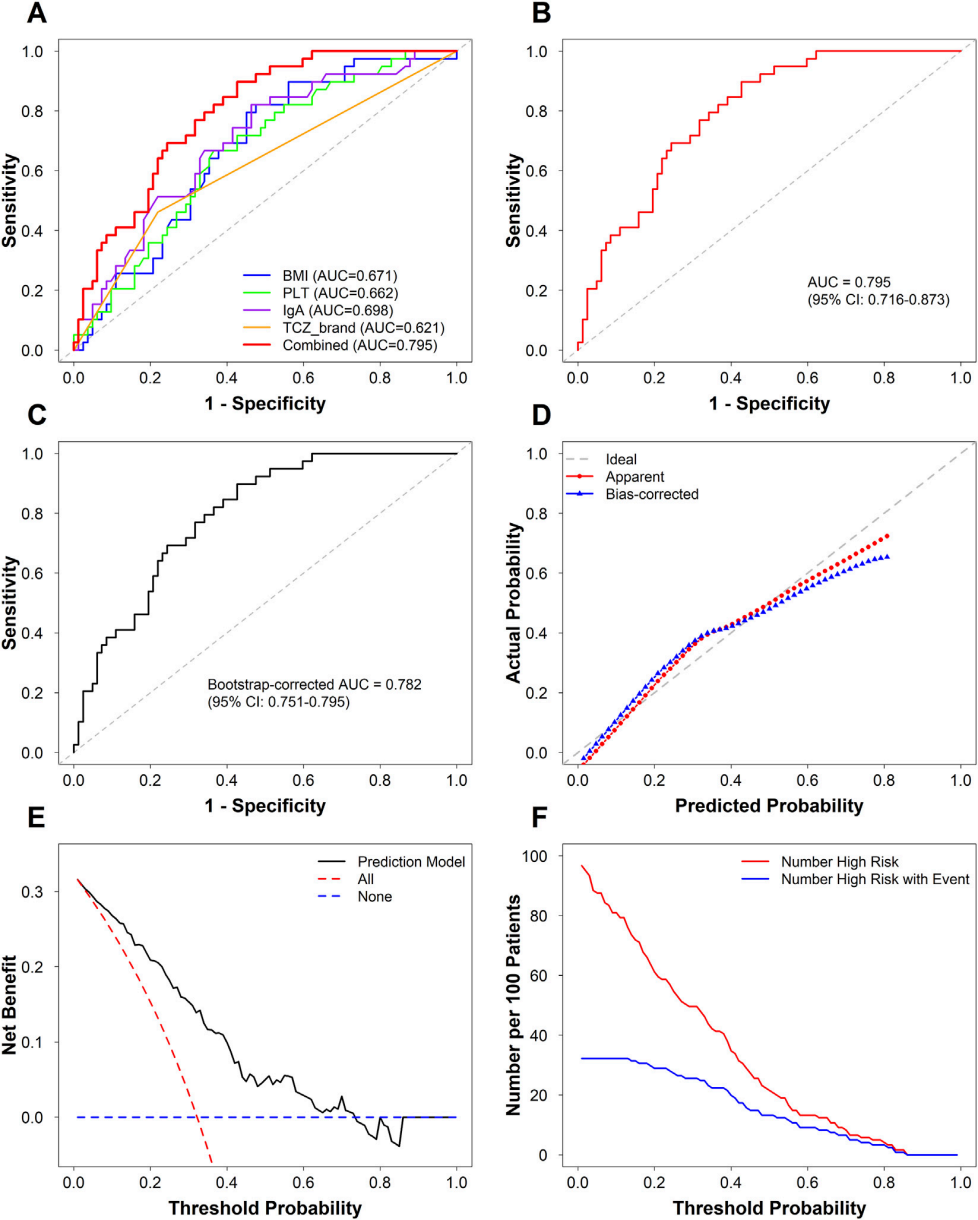
Model validation and clinical utility for predicting TCZ-associated hypofibrinogenemia. **(A)** Receiver operating characteristic curves of individual predictors and the combined model. **(B)** Receiver operating characteristic curve of the model. **(C)** Bootstrap-corrected receiver operating characteristic curve for internal validation. **(D)** Calibration curve. **(E)** Decision curve analysis. **(F)** Clinical impact curve.

The performance of the model was assessed in terms of discrimination, calibration and clinical utility. In the ROC analysis, the combined model showed an AUC of 0.795 (95% CI: 0.716–0.873), which was higher than the AUCs of the individual predictors BMI, PLT, IgA and TCZ brand, indicating better discrimination after integrating these variables (Figure 5A). Upon bootstrap internal validation (B = 1 000), the corrected AUC was 0.782 (95% CI: 0.751–0.795), confirming that the model retained stable discriminative performance after adjustment for overfitting (Figures 5B,C).

The DeLong test was performed to compare the apparent AUC with the bootstrap validated AUC. No significant difference was detected between the two estimates (*P* > 0.05), indicating no significant overfitting and supporting the stability of the model. Calibration analysis showed good agreement between predicted and observed probabilities and the bias corrected calibration curve remained close to the ideal reference line across most of the probability range (Figure 5D). This result was further supported by a Brier score of 0.170 and a corrected Brier score of 0.154, indicating acceptable overall predictive accuracy. In addition, the Hosmer Lemeshow test showed no significant deviation between predicted and observed outcomes (χ^2^ = 4.237, *P* = 0.835), further supporting good calibration.

DCA showed that the model provided a higher net benefit than the treat all and treat none strategies across a clinically relevant range of threshold probabilities, indicating potential value in clinical decision making (Figure 5E). The CIC also showed reasonable consistency between the number of patients classified as high risk and the number of true positive cases across relevant thresholds (Figure 5F). Overall, these findings suggest that the model has acceptable discrimination, good calibration, limited overfitting and potential clinical utility for identifying patients at increased risk of TCZ associated hypofibrinogenemia.

### 3.6 SHAP guided interpretation of the logistic regression model

**FIGURE 6:**
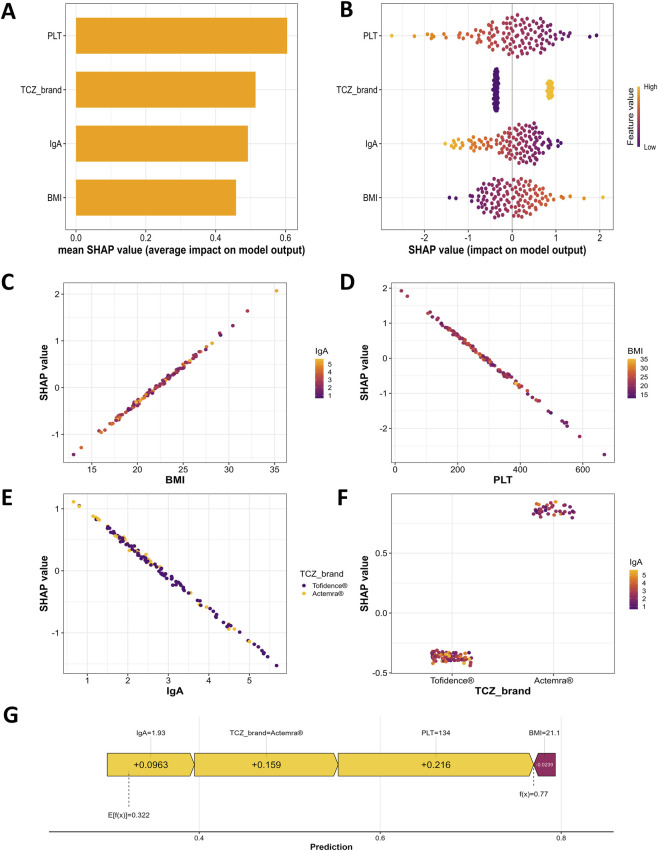
SHAP-based interpretation of the logistic regression model for TCZ-associated hypofibrinogenemia. **(A)** Global feature importance ranked by mean absolute SHAP values. **(B)** SHAP summary plot of predictor effects on model output. **(C–F)** Dependence plots illustrating the effects of body mass index, platelet count, age and drug brand on SHAP values. **(G)** SHAP force plot for an individual patient, showing the cumulative contribution of each predictor to the estimated risk.

To better elucidate the factors driving model predictions, SHAP analysis was conducted to quantify the relative importance and directional effects of the main predictors. Among all variables included in the model, PLT contributed most substantially to prediction, followed by TCZ brand, IgA and BMI (Figure 6A). These results suggest that treatment category and baseline patient characteristics were the primary determinants of the predicted risk of TCZ related hypofibrinogenemia.

The SHAP summary plot provided further information on the direction and magnitude of these effects (Figure 6B). Lower PLT values were associated with higher SHAP values, indicating an increased predicted risk of hypofibrinogenemia, whereas higher PLT values were associated with lower model output. Lower IgA levels corresponded to positive SHAP values, suggesting a higher predicted probability of this adverse event. In contrast, higher BMI corresponded to progressively higher SHAP values, supporting a positive association with risk. For TCZ brand, the two treatment categories showed clearly different contribution patterns, indicating a substantial difference in their influence on model prediction.

The SHAP dependence plots further demonstrated stable and biologically plausible relationships between these predictors and model output (Figures 6C–F). BMI showed an approximately linear positive association with SHAP value, indicating that the predicted risk increased as BMI rose (Figure 6C). PLT exhibited a clear inverse relationship with SHAP value, with lower platelet levels corresponding to higher predicted risk (Figure 6D). A similar inverse pattern was observed for IgA, showing that lower IgA levels were more likely to be classified as high risk (Figure 6E). For TCZ brand, the separation of SHAP values between categories remained distinct, confirming its independent discriminatory value in the model (Figure 6F).

At the individual level, the waterfall plot illustrated how these variables jointly shaped the final prediction (Figure 6G). In the representative case, TCZ brand (Actemra®), IgA level and PLT each contributed positively to the model output, while BMI exerted a minor negative contribution, collectively driving the predicted probability to 0.77. This finding highlights the interpretability of the model and indicates that the prediction was driven by transparent and clinically meaningful feature contributions.

Overall, these findings indicate that PLT, drug brand, IgA and BMI were the main predictors of TCZ related hypofibrinogenemia in the model. Specifically, lower PLT, lower IgA, higher BMI and a specific treatment category were associated with a higher predicted risk. Together, these results support the robustness of the model and reinforce its potential clinical utility in individualized risk assessment.

## 4 Discussion

We developed and validated a prediction model for TCZ-associated hypofibrinogenemia in patients with rheumatic diseases. The model demonstrated good discrimination, with an AUC of 0.795 (95% CI: 0.716–0.873) in the development cohort and a bootstrap-corrected AUC of 0.782 (95% CI: 0.751–0.795) after internal validation, indicating stable discriminative ability. We identified that higher BMI, lower IgA, lower PLT and the use of Actemra® were independent risk factors for the development of TCZ-associated hypofibrinogenemia in patients with rheumatic diseases. These findings add to the growing evidence on this under-recognized adverse drug reaction and provide new insights into risk stratification, suggesting the potential utility of our model as a tool to identify patients with rheumatic diseases at higher risk during TCZ treatment.

TCZ-associated hypofibrinogenemia occurred in 32.23% of patients (39/121), with a median time to onset of 64 days after treatment initiation. Which is consistent with the rates reported in recent real-world studies (An et al., 2024; Cai et al., 2025). Cai et al. (2025) reported a median onset time of 6 days, with 90.9% of cases occurring within 30 days of TCZ administration. In contrast, Yıldırım et al. found a median time to detection of hypofibrinogenemia of 9 months (range 1–108 months), with the earliest case occurring at 1 month (Yıldırım et al., 2024). In our cohort, despite a considerable proportion of patients developing TCZ-associated hypofibrinogenemia, no clinically significant bleeding events including ecchymosis, gingival bleeding, petechiae, hematemesis or other overt hemorrhagic manifestations were observed. This finding is consistent with the studies by An et al. (2024) and Souri et al. (2016), who also reported no bleeding symptoms in rheumatoid arthritis patients. However, Imamura et al. (2018) demonstrated that TCZ-treated patients had lower preoperative FIB levels and experienced greater blood loss after total knee arthroplasty compared with non-TCZ-treated patients, suggesting an increased bleeding risk in the surgical setting. Furthermore, a study by Cai et al. (2025) reported that bleeding occurred in patients with severe or life-threatening hypofibrinogenemia, significantly higher than in those with mild to moderate hypofibrinogenemia. These studies consistently demonstrate that hypofibrinogenemia is not a rare event during TCZ therapy, underscoring the need for routine monitoring, particularly in high-risk patients.

Our study found that higher BMI, lower IgA, lower PLT and the use of Actemra® were independent risk factors for the development of TCZ-associated hypofibrinogenemia in patients with rheumatic diseases. The risk factors in our study differ substantially from those reported in the literature, highlighting the heterogeneity of patient populations and study designs. In the largest retrospective study to date, Cai et al. identified infection, COVID-19, CAR-T therapy and concomitant glucocorticoids as independent risk factors, whereas high baseline FIB level and concomitant antirheumatic drugs were protective. Our results showed no significant differences in concomitant glucocorticoids or concomitant antirheumatic drugs between the two groups. The inflammatory milieu and consumption coagulopathy in acute settings may differ fundamentally from that in chronic autoimmune diseases, leading to distinct risk profiles. An et al. focused specifically on rheumatoid arthritis patients and reported that platelet distribution width, parathyroid hormone, bone mineral density, tender joint count, and swollen joint count were independent risk factors for TCZ-associated hypofibrinogenemia (An et al., 2024). Unlike the study by An et al., we also collected rheumatology-specific variables including tender joint count, swollen joint count, platelet distribution width, parathyroid hormone, and bone mineral density in our baseline assessment. However, these variables did not show significant differences between the hypofibrinogenemia and control groups in univariate analysis. Our study identified low baseline serum IgA levels as an independent risk factor for tocilizumab-induced hypofibrinogenemia. Low baseline IgA may reflect a blunted IL-6/gp130/STAT3 signaling pathway, which drives both hepatic fibrinogen synthesis and IgA class switching (Gauldie et al., 1987; Cerutti, 2008). When this pathway is further blocked by tocilizumab, fibrinogen production becomes more vulnerable to decline. Additionally, IgA deficiency is often associated with impaired intestinal barrier function and endotoxin translocation, which may interfere with hepatic protein synthesis and promote fibrinogen consumption (Levi and van der Poll, 2010). We also observed that higher PLT was associated with a reduced risk of hypofibrinogenemia. PLT and FIB play closely related roles in hemostasis and both are acute-phase reactants that increase in response to inflammation. Additionally, thrombocytopenia has been reported as a concurrent adverse event in some cases of TCZ-associated hypofibrinogenemia (Vitiello et al., 2017). Our findings suggest that patients with lower PLT may be more vulnerable to hypofibrinogenemia and close monitoring of both parameters is advisable. A novel finding of our study is that the use of Actemra® (reference TCZ) was associated with a more than threefold increased risk of hypofibrinogenemia compared to Tofidence® (OR: 3.423). Although no previous study has compared this hypofibrinogenemia risk across different TCZ products, recent analyses of the biosimilar BAT1806/BIIB800 (Tofidence®) offer potential explanations. Structurally, BAT1806/BIIB800 has a higher glycation content (10.08% vs. 1.19% for reference TCZ) but similar IL-6R binding and effector functions (Liu et al., 2025); whether this affects FIB metabolism is unknown. Immunologically, the biosimilar elicited a higher anti-drug antibody rate (21% vs. 14%) (Leng et al., 2024), which might accelerate drug clearance and attenuate the fibrinogen-lowering effect. Our finding underscores the need for pharmacovigilance to determine whether product-specific differences influence the safety profile of TCZ.

The discrepancies in identified risk factors between our study and previous reports likely stem from several key differences. First, Cai et al. studied predominantly acute inflammatory conditions (COVID-19, CAR-T therapy) (Cai et al., 2025), where hypofibrinogenemia is driven by consumption coagulopathy superimposed on IL-6 blockade, whereas our chronic rheumatic cohort reflects gradual FIB decline following IL-6 inhibition—fundamentally different pathophysiological contexts. Second, An et al. focused exclusively on RA (An et al., 2024), while we included mixed rheumatic diseases; this broader population may have diluted disease-specific signals but also increased generalizability. Finally, our use of LASSO regression for variable selection prior to multivariable modeling may retain different predictors compared with conventional stepwise approaches used in other studies. Collectively, these differences suggest that the risk profile of TCZ-associated hypofibrinogenemia is context-dependent, and our findings should be viewed as complementary to, rather than contradictory of, prior work. Given the relatively high incidence of hypofibrinogenemia and its potential to cause bleeding, particularly in severe cases, routine monitoring of fibrinogen levels during TCZ therapy is essential. Our results suggest that closer monitoring should be considered in patients with lower IgA, those with higher BMI, lower PLT and those receiving the Actemra®. A risk prediction model incorporating these factors could help clinicians identify high-risk patients and implement preventive strategies, such as more frequent laboratory monitoring or judicious use of concomitant anticoagulants.

There are several limitations in our study. First, this retrospective, single-center study had a relatively small sample size, with only 39 events among 121 patients. The limited number of events restricted our ability to include more candidate variables in the multivariable model, and the risk of overfitting cannot be excluded despite internal validation. Second, reliance on existing medical records constrained standardized data collection and precluded longitudinal assessment. Third, the prediction model showed only moderate discrimination (AUC = 0.795) and lacked external validation; therefore, its accuracy and generalizability to other populations remain unknown. Fourth, the observed brand effect (Actemra®) should be interpreted with caution because brand use was largely determined by hospital procurement policies and reimbursement changes, leading to near-complete temporal separation between brands. Thus, the brand effect may partly reflect period-related confounders rather than true biological differences. Finally, despite efforts to adjust for potential confounders, unmeasured or residual confounding inherent to retrospective designs cannot be ruled out. Future prospective, multicenter studies with larger cohorts are warranted to validate our findings.

## 5 Conclusion

In summary, our study identified that lower IgA, higher BMI, lower PLT and use of Actemra® are independent risk factors for TCZ-associated hypofibrinogenemia in patients with rheumatic diseases. These findings extend the existing literature and may help guide risk stratification and monitoring strategies. Further research is needed to elucidate the underlying mechanisms and to determine whether product-specific differences in TCZ formulations affect the safety profile.

## Data Availability

Due to ethical restrictions, the de-identified data underlying this study are not publicly available. They can be obtained from the corresponding author (Xiaoyan Li) on reasonable request, subject to institutional ethics approval and a signed data access agreement. Requests to access these datasets should be directed to Xiaoyan Li (lixyan5@mail.sysu.edu.cn).
