# Supplementary material for: A nomogram based on LASSO-logistic regression for predicting tocilizumab-associated hypofibrinogenemia in patients with rheumatic diseases
Source: Front Pharmacol. 2026 Jul 31;17:1861187. doi: 10.3389/fphar.2026.1861187 (PMC13473360; doi:10.3389/fphar.2026.1861187)
Supplement: Supplementary file 1 [file Supplementaryfile1.docx]

Supplementary Material

**Supplementary Figure S1.** Distribution of missing data among the clinical and laboratory variables.

**Supplementary Table S1 Missing data summary for continuous variables before and after imputation**

| **Variable** | **Missing before imputation, n (%)** | **Missing after imputation, n (%)** |
| --- | --- | --- |
| GGT | 28 (23.1%) | 0 (0.0%) |
| INR | 6 (5.0%) | 0 (0.0%) |
| APTT | 6 (5.0%) | 0 (0.0%) |
| TT | 6 (5.0%) | 0 (0.0%) |
| FIB | 6 (5.0%) | 0 (0.0%) |
| TC | 6 (5.0%) | 0 (0.0%) |
| TG | 6 (5.0%) | 0 (0.0%) |
| TBIL | 5 (4.1%) | 0 (0.0%) |
| IgG | 4 (3.3%) | 0 (0.0%) |
| IgA | 4 (3.3%) | 0 (0.0%) |
| IgM | 4 (3.3%) | 0 (0.0%) |
| C3 | 4 (3.3%) | 0 (0.0%) |
| C4 | 4 (3.3%) | 0 (0.0%) |
| RF | 3 (2.5%) | 0 (0.0%) |
| Anti-CCP | 3 (2.5%) | 0 (0.0%) |
| Tender joint count | 1 (0.8%) | 0 (0.0%) |
| Swollen joint count | 1 (0.8%) | 0 (0.0%) |
| BMI | 1 (0.8%) | 0 (0.0%) |
| ESR | 1 (0.8%) | 0 (0.0%) |
| Cr | 1 (0.8%) | 0 (0.0%) |
| Disease duration | 0 (0.0%) | 0 (0.0%) |
| Drug dosage | 0 (0.0%) | 0 (0.0%) |
| Age | 0 (0.0%) | 0 (0.0%) |
| WBC | 0 (0.0%) | 0 (0.0%) |
| RBC | 0 (0.0%) | 0 (0.0%) |
| HGB | 0 (0.0%) | 0 (0.0%) |
| PLT | 0 (0.0%) | 0 (0.0%) |
| CRP | 0 (0.0%) | 0 (0.0%) |
| ALB | 0 (0.0%) | 0 (0.0%) |
| ALT | 0 (0.0%) | 0 (0.0%) |
| AST | 0 (0.0%) | 0 (0.0%) |
| GLU | 0 (0.0%) | 0 (0.0%) |

FIB, fibrinogen; BMI, body mass index; WBC, White Blood Cell count; RBC, red blood cell count; HGB, hemoglobin; PLT, platelet count; CRP, C-reactive protein; ESR, erythrocyte sedimentation rate; RF, rheumatoid factor; Anti-CCP, anti-cyclic citrullinated peptide antibody; APTT, activated partial thromboplastin time; TT, thrombin time; INR, international normalized ratio; TBIL, total bilirubin; TG, Triglycerides; TC, total cholesterol; GGT, gamma-glutamyl transferase; ALB, albumin; ALT, alanine aminotransferase; AST, aspartate aminotransferase; Cr, creatinine; IgG, immunoglobulin G; IgA, immunoglobulin A; IgM, immunoglobulin M; C3, complement C3; C4, complement C4; GLU, glucose.

**Supplementary Table S2 Regression coefficients from LASSO Model at lambda.1se**

| **Variables** | **Coefficient** |
| --- | --- |
| BMI | 0.011 |
| PLT | -0.001 |
| TCZ brand (Actemra®) | 0.176 |
| TG | 0.07 |
| IgA | -0.174 |

TCZ, tocilizumab; IgA, immunoglobulin A; BMI, body mass index; PLT, platelet count; TG, triglyceride.
